# Changes in Toxin Production, Morphology and Viability of *Gymnodinium catenatum* Associated with Allelopathy of *Chattonella marina* var. *marina* and *Gymnodinium impudicum*

**DOI:** 10.3390/toxins14090616

**Published:** 2022-09-03

**Authors:** Leyberth José Fernández-Herrera, Christine Johanna Band-Schmidt, Tania Zenteno-Savín, Ignacio Leyva-Valencia, Claudia Judith Hernández-Guerrero, Francisco Eduardo Hernández-Sandoval, José Jesús Bustillos-Guzmán

**Affiliations:** 1 Instituto Politécnico Nacional, Centro Interdisciplinario de Ciencias Marinas (IPN-CICIMAR), Av. Instituto Politécnico Nacional s/n, Col. Playa Palo de Santa Rita, La Paz C.P. 23096, BCS, Mexico; 2 Centro de Investigaciones Biológicas del Noroeste (CIBNOR), Instituto Politécnico Nacional 195, Col. Playa Palo Santa Rita Sur, La Paz C.P. 23096, BCS, Mexico; 3 Consejo Nacional de Ciencia y Tecnología-Instituto Politécnico Nacional, Centro Interdisciplinario de Ciencias Marinas (CONACyT, IPN-CICIMAR), Col. Playa Palo de Santa Rita, La Paz C.P. 23096, BCS, Mexico

**Keywords:** allelochemical, chemical ecology, paralytic shellfish toxins

## Abstract

Allelopathy between phytoplankton organisms is promoted by substances released into the marine environment that limit the presence of the dominating species. We evaluated the allelopathic effects and response of cell-free media of *Chattonella* *marina* var. *marina* and *Gymnodinium impudicum* in the toxic dinoflagellate *Gymnodinium catenatum*. Additionally, single- and four-cell chains of *G. catenatum* isolated from media with allelochemicals were cultured to evaluate the effects of post exposure on growth and cell viability. Cell diagnosis showed growth limitation and an increase in cell volume, which reduced mobility and led to cell lysis. When *G. catenatum* was exposed to cell-free media of *C. marina* and *G. impudicum*, temporary cysts and an increased concentration of paralytic shellfish toxins were observed. After exposure to allelochemicals, the toxin profile of *G. catenatum* cells in the allelopathy experiments was composed of gonyautoxins 2/3 (GTX2/3), decarcarbamoyl (dcSTX, dcGTX2/3), and the sulfocarbamoyl toxins (B1 and C1/2). A difference in toxicity (pg STXeq cell^−1^) was observed between *G. catenatum* cells in the control and those exposed to the filtrates of *C. marina* var. *marina* and *G. impudicum.* Single cells of *G. catenatum* had a lower growth rate, whereas chain-forming cells had a higher growth rate. We suggest that a low number of *G. catenatum* cells can survive the allelopathic effect. We hypothesize that the survival strategy of *G. catenatum* is migration through the chemical cloud, encystment, and increased toxicity.

## 1. Introduction

Cell viability and survival of phytoplankton species suffering from allelopathy is low [1,2]. Allelopathy is defined as a biochemical interaction in which a donor species produces one or more chemical compounds that can affect the growth of similar target species [3]. In some interactions, allelopathy includes any direct or indirect, negative or positive, consequence of chemical substances secreted by plants and microorganisms [4]. Allelopathy in phytoplankton includes only the negative effects promoted by the donor species through the production of chemical compounds called allelochemicals on the target species [5,6,7,8]. Allelopathic donor species cause high mortalities in phytoplankton species that suffer negative allelopathic effects due to damage to photosynthetic efficiency, oxidative stress, inhibition of enzymatic activity, and damage to nucleic acids, all of which are reflected in the loss of cell mobility, morphological changes, changes in osmoregulation, and the formation of temporary cysts, as well as cell membrane disruption and lysis [5,6]. However, these responses may differ depending on the competitors involved [9,10,11,12,13]. Harmful algal-bloom-forming ichthyotoxic microalgae have been reported to have the ability to promote or undergo allelopathy [14,15]. For instance, *Margalefidinium polykrikoides* (Margalef) F.Gómez, Richlen & D.M.Anderson has an allelopathic effect on several species of different planktonic groups [16,17], and *Akashiwo sanguinea* (K.Hirasaka) Gert Hansen & Moestrup, inhibits the growth of co-occurring phytoplankton species, including *Scrippsiella trochoidea* (F. Stein) A.R.Loeblich III, =*Scrippsiella acuminata* (Ehrenberg) Kretschmann, Elbrächter, Zinssmeister, S.Soehner, Kirsch, Kusber & Gottschling, *Phaeocystis globosa* Scherffel, and *Rhodomonas salina* (Wisłouch) D.R.A.Hill & R.Wetherbee [18]. The diatom *Skeletonema costatum* (Greville) Cleve can reduce the growth and affect several metabolic processes in the ichthyotoxic dinoflagellate *Karenia mikimotoi* (Oda) Gert Hansen & Moestrup [6].

Under laboratory conditions, *M. polykrikoides, Gymnodinium impudicum* (S.Fraga & I.Bravo) Gert Hansen & Moestrup and *Chattonella marina* var. *marina* (Subrahmanyan) Y.Hara & M.Chihara can dominate the paralytic shellfish toxin (PST) producer dinoflagellate *Gymnodinium catenatum* H.W.Graham [17,19,20]. However, production of PST in response to the allelopathic effect is unknown. *Chattonella marina* produces and releases polyunsaturated fatty acids and reactive oxygen species into the marine environment [21,22,23,24], whereas *G. impudicum* produces mucilage formed by exopolysaccharides [25,26]. During the early exponential phase, *G. impudicum* can produce superoxide radicals in similar concentrations to those of *C. marina* var. *marina* [17]. These compounds have been related to ichthyotoxic and allelopathic activity and identified as signaling molecules in phytoplankton intra/interspecific interactions [16,27].

The complexity of the allelopathy phenomenon in the community structure eventually allows for the donor species to become a target species. For example, *M. polykrikoides*, *A. sanguinea*, and *C. marina* are growth-limited by the allelopathic effect of the ichthyotoxic dinoflagellate *Alexandrium leei* Balech [28]. The pathways that modulate the community dynamics through allelopathy make it a complex phenomenon. Furthermore, biotic and abiotic factors influence the release of substances into the environment, making it difficult to study them in the field. This complexity highlights the fact that most knowledge with respect to the mechanisms of action and response of allelopathy in phytoplankton (chlorophytes, diatoms, cyanobacteria, and dinoflagellates) has been acquired under laboratory conditions [29,30,31]. The intensity of the allelopathic interaction depends on the abundance and the cellular contact between the co-occurring species; most studies have been focused on demonstrating the effects on the target species and the identification of the allelochemicals responsible for the allelopathic effect [32,33,34]. Allelopathy between co-occurring phytoplankton species is a determining factor in the community structure and in the succession of microalgae species in the marine environment [35,36,37]. The production of allelochemicals is considered a strategy to limit or eliminate competitors or to survive the presence of predators [38,39]. Such strategies involve allelopathy as a determining factor in a coevolutionary context of donor species towards the survival response of the dominated species [6,32,40,41,42], suggesting that the responses and strategies that are activated in target species can contribute to understanding how phytoplankton groups restructure the community after being temporarily severely minimized during the dynamic composition of the phytoplankton community and during the formation of harmful algal blooms (HABs) [43,44,45,46]. However, in most cases, the mechanism responsible for the responses to allelopathic compounds is unknown.

To document the changes undergone by *G. catenatum* during allelopathy, we evaluated its response to the effects caused by cell-free media from *C. marina* var. *marina* and *G. impudicum* under laboratory conditions. Changes in PST profile and contents, growth, abundance, volume, and cell morphology were determined. In addition, the cell viability of single cells and four-cell chains of *G. catenatum* after exposure to cell-free media was evaluated.

## 2. Results

### 2.1. Allelopathy Experiment: Changes in Cell Shape and Volume

**Figure 1:**
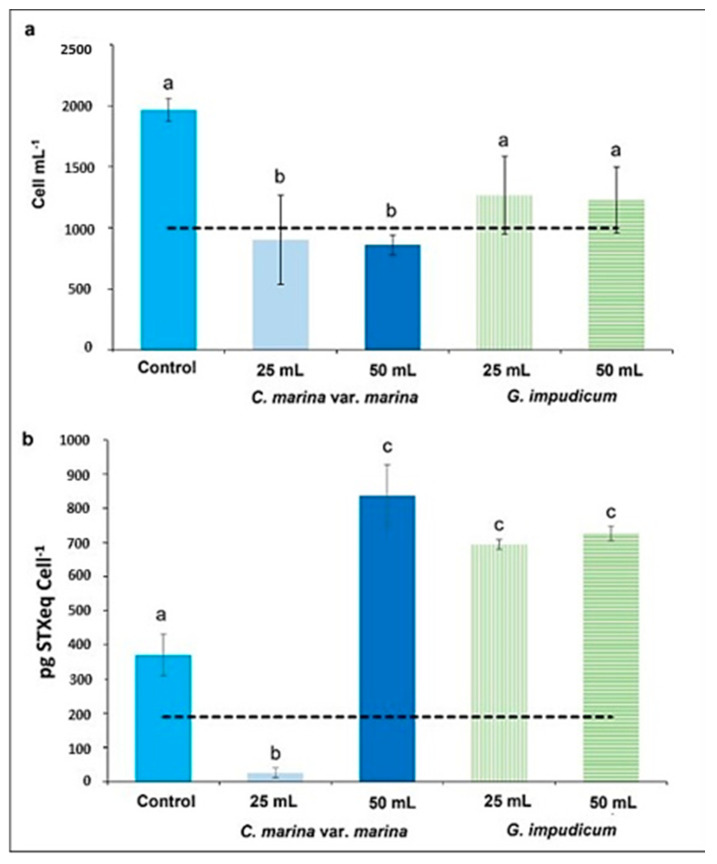
(**a**) *Gymnodinium catenatum* cell abundance after 48 h of allelopathy exposure to 25 and 50 mL cell-free media of *Chattonella marina* var. *marina* and *Gymnodinium impudicum*. The dotted line represents the initial cell abundance and toxicity. (**b**) Average toxicity per cell of *G. catenatum* after 48 h of allelopathy exposure to 25 and 50 mL cell-free media from *C. marina* var. *marina* and *G. impudicum*. Data are show as mean ± SD. Different letters mark significant differences between the treatments; the same letters indicate no statistical differences between treatments (one-way ANOVA, *p* < 0.05, *n* = 3).

The resultant cell abundance of *G. catenatum* after 48 h of exposure to cell-free media of *C. marina* var. *marina* and *G. impudicum* is shown in Figure 1a. Exposure to cell-free media from *C. marina* var. *marina* caused mortality, whereas cell-free media of *G. impudicum* promoted an increase in the growth of *G. catenatum*, although below the control value. After 48 h of exposure to 25 mL and 50 mL of the filtrate of *C. marina* var. *marina*, mortality was 10 ± 1 and 14 ± 3%, reaching a final cell abundance of 902 ± 63 cells mL^−1^ and 860 ± 69 cells mL^−1^, respectively (ANOVA, *p* = 0.018, *p* = 0.013). Cell abundance was significantly lower than in the control, increasing by 95 ± 12%, from 1000 ± 31 cells mL^−1^ to 1966 ± 94 cells mL^−1^. With 25 and 50 mL cell-free media of *G. impudicum*, the final cell abundance of *G. catenatum* reached similar values: 1268 ± 316 and 1229 ± 270 cells mL^−1^, respectively. This represents 36 ± 16 and 37 ± 13% less than in the control (NS, ANOVA, *p* = 0.175; *p* = 0.141).

The abundance in the single cells in the control was 15.98 ± 2.65%, that of two-cell chains was 37.46 ± 2.00%, that of four-cell chains was 39.79 ± 4.47%, and that of eight-cell chains was 6.77 ± 0.16%. The percentage of chain cells was highest in *G. catenatum* following exposure to cell-free media from *C. marina* var. *marina* and *G. impudicum*. Following exposure to 25 mL of cell-free media from *C. marina* var. *marina*, 63.64 ± 17.65% corresponded to two-cell chains. With 50 mL of *C. marina* var. *marina* cell-free media, 52.32 ± 32% of cells were found to form four-cell chains, whereas 25 and 50 mL of cell-free media from *G. impudicum* resulted in 55.31 ± 7.96 and 48.37 ± 4.33% cells formed by four-cell chains, with significant differences in all cases between the control and treatment groups (one-way ANOVA, *p* < 0.05) (Figure S1).

A difference in toxicity (pg STXeq cell^−1^) was found between *G. catenatum* cells in the control group and those exposed to the filtrates of *C. marina* var. *marina* and *G. impudicum* (Figure 1b). Exposure to 25 and 50 mL of the cell-free medium of *C. marina* var. *marina* caused a decrease (26.16 ± 14.31) and increase (836 ± 90.42) in toxicity, respectively, compared with the control (370 ± 61.47), with significant differences (one-way ANOVA, *p* = 4.7^−4^). When *G. catenatum* was exposed to cell-free media of *G. impudicum*, an increase in toxicity was observed in both treatments, with 693 ± 14.58 pg STXeq cell^−1^ and 726 ± 21.84 pg STXeq cell^−1^ for 25 and 50 mL cell-free media additions, respectively. These treatments were statistically different from the control (one-way ANOVA, (*p* = 7.2^−4^) and (*p* = 3.9^−4^), respectively) in terms of toxic content (pg cell^−1^) per saxitoxin analog (Table S1).

**Figure 2:**
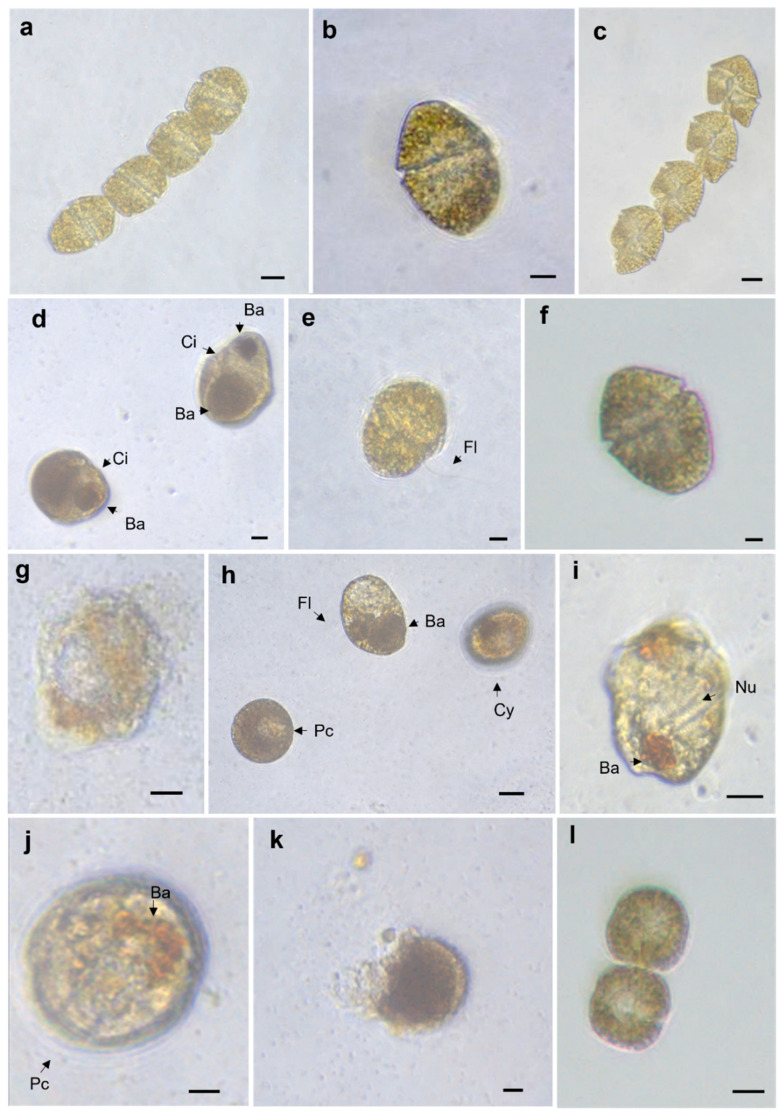
Micrographs of *Gymnodinium catenatum*. Control treatment: (**a**) four cell-chain, (**b**) lateral view of a single cell, and (**c**) four cell-chain in division. Cells of *G. catenatum* exposed to 25 mL of *Chattonella marina* var. *marina* cell-free media: (**d**) two deformed cells with an increase in size, (**e**) deformed cell with flagellum, and (**f**) a cell resembling a planomeiocyte. Cells of *G. catenatum* exposed to 50 mL of *C. marina* var. *marina* cell-free media: (**g**) lysis of a temporary cyst, (**h**) cell with flagella with deformation, and two temporary cysts in formation. (**i**) Detail of cytoplasm and orange-brown accumulation body, nucleus, and chromosomes. *G. catenatum* exposed to 25 mL of *Gymnodinium impudicum* cell-free media: (**j**) temporary cyst and (**k**) temporary cyst in lysis. *G. catenatum* exposed to 50 mL of *Gymnodinium impudicum* cell-free media: (**l**) two deformed chain-forming cells. Accumulation body (**ab**), cingulum (**ca**), sulcus (**su**), flagella (**fl**), cyst (**cy**), pellicle cyst (**pc**), nucleus (**nu**), pyrenoid (**py**). Scale bar: 30 μm.

The chain-forming cells in the control group were slightly round and elongated rather than wide, with a well-defined sulcus and multiple plastids (Figure 2a). Single cells had a well-defined conical, epicone and a trapezoidal hypocone with visible pyrenoids (Figure 2b). Cells in division had a wide and slightly displaced sulcus at the cingulum; in some cells, the nucleus was observed in a central position, and multiple small chloroplasts were visible (Figure 2c). Cell-free media from *C. marina* var. *marina* and *G. impudicum* caused changes in the morphology of *G. catenatum*, such as rounded cells with multiple vacuolation, condensation of the cytoplasm with accumulation bodies (Figure 2c), a faintly observed cingulum and sulcus (Figure 2d), loss/disintegration of the cell membrane, loss of the longitudinal flagellum (Figure 2e), a rounded epicone and hypocone (Figure 2f), and lysis (Figure 2g). When cell-free media of *G. impudicum* was added, cytoplasm condensation with multiple accumulation bodies was observed (Figure 2h,i). A total loss of the typical morphology of *G. catenatum* occurred, but cells maintained the longitudinal flagellum (Figure 2h). Temporary cysts were also observed; when reisolated, none of the temporal cysts were viable during the observation time of the experiment (Figure 2h,j), and cysts lysed (Figure 2k). In the treatments with cell-free media of *C. marina* var. *marina* and *G. impudicum* (see video S1), rounded cells with rough membranes and cell elongations, as well as evident nuclei, were found recurrently (Figure 2i,j), in addition to large cells resembling a planozygote (Figure 2f) and the formation of rare temporary cysts (Figure 2i).

Rare temporary cyst formation was recorded only in the allelopathy treatments; 13 temporary cysts of *G. catenatum* were recorded after exposure to 25 mL of *C. marina* var. *marina* cell-free media, and 10 were recorded following exposure to 50 mL of cell-free media. In the treatment with *G. impudicum*, 16 and 19 temporary cysts of *G. catenatum* were recorded with 25 and 50 mL cell-free media exposure, respectively.

With respect to the morphological changes in *G. catenatum*, cells exposed to cellular filtrates increased in size relative the control cells (Table 1). When *G. catenatum* was exposed to 25 and 50 mL of cell-free media from *C. marina* var. *marina*, cells had an average width of 46.58 ± 10.91 µm and 45.34 ± 0.99 µm, respectively, with an average cell length of 52.04 ± 15.30 µm and 51.91 ± 15.78 µm, respectively. Cells were significantly larger, with an average volume of 27,495 ± 9388 µm^3^, than cells in the control treatment group, with an average volume of 19,550 ± 8316 µm^3^ (ANOVA, *p =* 0.001). Cellular filtrates of *G. impudicum* also caused an increase in cell size in *G. catenatum*, the cells of which were larger than cells in the control treatment group, with an average width of 46.43 ± 11.01 µm and an average length of 53.20 ± 17.29 µm when exposed to 25 mL of cell-free media. When exposed to 50 mL of *G. impudicum* cell-free media, cells of *G. catenatum* had a width of 46.47 ± 10.78 µm and a length of 52.17 ± 18.28; the average volume in both treatments was similar: ~25,995 ± 9388 µm^3^. No significant differences were found in terms of volume between cells exposed to cell-free filtrates of *C. marina* var. *marina* (ANOVA, *p =* 0.986) and the control group (ANOVA, *p =* 0.173). No numerical or statistically significant differences were found between the two control treatments. Therefore, for practical purposes, the average of both treatments is shown in graphs and tables as a single control.

### 2.2. Paralytic Toxin Profile

**Figure 3:**
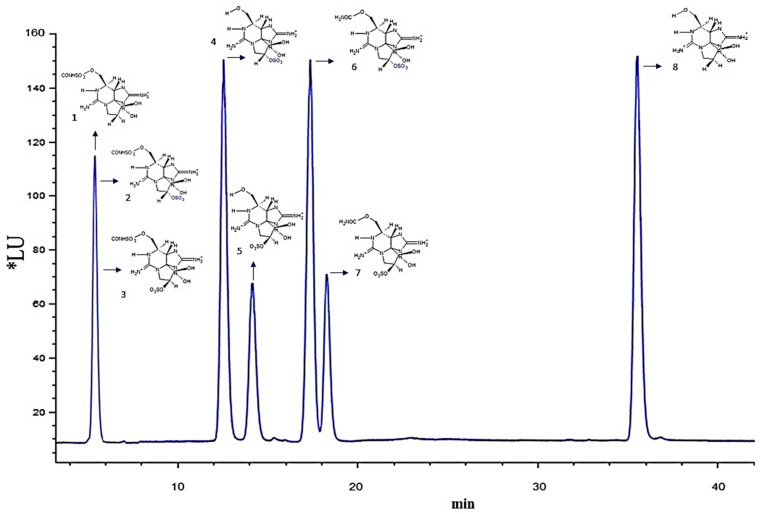
Chromatograms of toxin standards. Sulfocarbamoyl: 1–3 (B1, C1/2), biotransformation product through a hydrolysis reaction. Decarbamoyl: 4 (dcGTX2), 5 (dcGTX3), 8 (dcSTX). Carbamoyl: 6 (GTX2), 7 (GTX3). Y axis: luminescence unit (*LU); X axis: retention time (min); excitation wavelength: 330 nm; emission wavelength: 395 nm.

**Figure 4:**
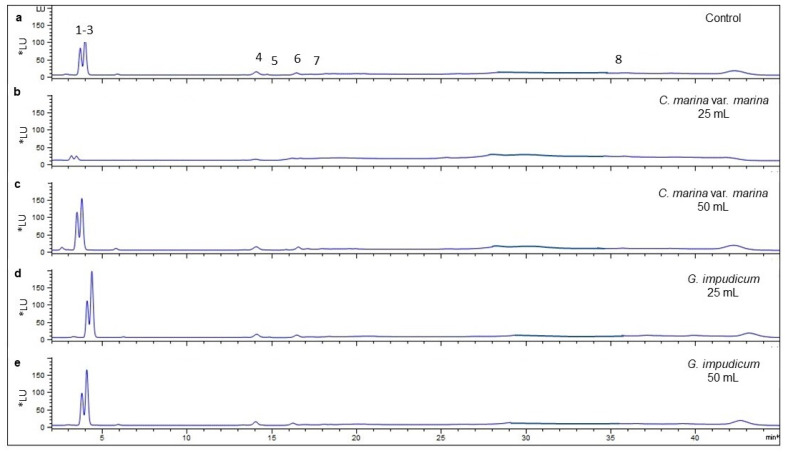
Chromatograms of the toxin profile of *Gymnodinium catenatum.* (**a**) Control after allelopathy exposure to cell-free media from *Chattonella marina* var. *marina* ((**b**) 25 mL, (**c**) 50 mL) and *Gymnodinium impudicum* ((**d**) 25 mL and (**e**) 50 mL). Sulfocarbamoyl: 1–3 (B1,C1/2), biotransformation product through a hydrolysis reaction. Decarbamoyl: 4 (dcGTX2), 5 (dcGTX3), 8 (dcSTX). Carbamoyl: 6 (GTX2), 7 (GTX3). Y axis: luminescence unit (*LU); X axis: retention time (min); excitation wavelength: 330 nm; emission wavelength: 395 nm.

The toxin profile in *G. catenatum* cells in the allelopathy experiments comprised carbamoyl gonyautoxins 2/3 (GTX2/3), decarcarbamoyl (dcSTX, dcGTX2/3), and sulfocarbamoyl toxins (B1 and C1/2). Chromatograms of the toxin profiles and toxin standards are shown in Figure 3 and Figure 4 and Table 2.

In the control treatment, the average molar percentage of the analogs was 1.43 ± 0.26% GTX2/3, 3.38 ± 0.21% decarbamoyl toxins, and 94.44 ± 0.48% sulfocarbamoyl toxins (B1 and C1/2). When 25 mL of cell-free media from *C. marina* var. *marina* was added, 5.06 ± 0.41% GTX2/3 was detected, decreasing to 1.48 ± 0.06% with a volume of 50 mL of cell-free media, with no statistically significant differences relative to the control (Kruskal–Wallis, *p* = 0.730). For the decarbamoyl analogs, the molar percentage was 7.00 ± 0.43% when exposed to 25 mL of cell-free media, decreasing to 2.91 ± 0.16% with significant differences relative to the control and with 50 mL *C. marina* var. *marina* and 25–50 mL of *G. impudicum* cell-free media treatments (one-way, ANOVA, *p* < 0.05). When 50 mL cell-free media from *C. marina* var. *marina* was added, statistically significant differences relative to the control and *G. impudicum* treatments were observed (one-way ANOVA, *p* < 0.05). The sulfocarbamoyl toxin concentrations (B1 and C1/2) corresponded to 85.84 ± 3.46% when *G. catenatum* was exposed to 25 mL of cell-free media from *C. marina* var. *marina*, with statistically significant differences relative to the control and 50 mL cell-free media from *G. impudicum* (Kruskal–Wallis, *p =* 0.025), and 94.96 ± 0.11% of C1/2 toxin was detected following the addition of 50 mL cell-free media from *C. marina* var. *marina*, although no statistically significant differences were observed relative to the control and the other treatments (Kruskal–Wallis, *p* > 0.05) (average (% mol) per type of saxitoxin analogs in Table S2).

### 2.3. Viability, Growth Rate, and Generation Time

#### 2.3.1. Single Cell

Individual *G. catenatum* cells had a lower viability percentage than the control compared to four-cell chains when exposed to the cell-free media. In the control treatment, 46 ± 2% of the (50) wells containing individual cells had live and dividing cells. Only 14 ± 4% of the wells containing individual cells exposed to 25 mL of *C. marina* var. *marina* had live cells, whereas only 8 ± 3% of wells exposed to 50 mL cell-free media had live cells. These values were 69.6% and 82.6 ± 2% less than the control, respectively (Table 3). In viable wells with cells isolated from the cell-free filtrate of *C. marina* var. *marina*, dividing cells of *G. catenatum* were also observed. Survival of individual cells of *G. catenatum* reisolated from the treatment exposed to 25 mL of cell-free media from *G. impudicum* occurred in 24 ± 6% of the wells, and only 47.9% of the wells had live cells relative to the control. When exposed to 50 mL of cell-free filtrates of *G. impudicum*, 20 ± 3% of the wells with *G. catenatum* cells were viable, i.e., 56.6% less than the control, and cell division was observed in wells with live cells (Table 3).

The growth rate of individual cells reisolated from the controls was higher than that of *G. catenatum* cells exposed to cell-free media (Table 3). The control had a growth rate of 1.57 ± 0.28 div day^−1^, which was three times higher than that of cells reisolated following exposure to 25 mL of cell-free media of *C. marina* var. *marina* and *G. impudicum*, with a growth rate of 0.55 ± 0.33 and 0.45 ± 0.33 div day^−1^, respectively (Kruskal–Wallis test*, p* = 0.021, *p* = 0.010). Cells reisolated from treatments exposed to 50 mL of cell-free media of *C. marina* and *G. impudicum* had a higher growth rate (1.03 ± 0.49 div day^−1^ and 0.90 ± 0.17 div day^−1^, respectively) than the control; in neither case was there a significant difference relative to the control treatment (*p* = 0.605). However, there was a significantly higher growth rate (Kruskal–Wallis, *p* = 0.002) when a higher volume of cell free filtrate was added.

*Gymnodinium catenatum* cells isolated from the control group had a generation time of 0.14 ± 0.03 day^−1^, similar to the generation time of cells exposed to 25 mL filtrates of *C. marina* var. *marina* (0.16 ± 0.2 day^−1^). Single cells reisolated from the treatments with 50 mL of *C. marina* var. *marina* cell-free media had a significantly longer generation time of 0.32 ± 0.15 day^−1^ (Kruskal–Wallis, *p* = 0.005). The addition of 25 and 50 mL of *G. impudicum* cell-free media *G. catenatum* resulted in a significantly longer generation time of ~0.31 ± 0.10 day^−1^ compared to the control and the treatment with 25 mL of *C. marina* var. *marina* (Kruskal–Wallis, *p* = 0.005) (Table 3).

#### 2.3.2. Four-Cell Chains

In chain-forming cells, the increased viability (62 ± 3%) of the seeded wells was observed in the treatment with 25 mL of cell-free media from *C. marina* var. *marina,* which was 13 ± 1% lower with respect to the control. Growth in cells exposed to 50 mL of cell-free media of *C. marina* 28 ± 3% was 54.9 ± 4% lower than in the control. When chains of *G. catenatum* were reisolated from 25 mL of the cell-free medium of *G. impudicum*, 50 ± 7% of the wells were viable—exactly half as many as in the control—whereas in the treatment with 50 mL of cell-free media, 41 ± 9% of the wells with *G. catenatum* cells were viable—33.9 ± 3% less than in the control (Table 4).

The growth rate of four-cell chains of *G. catenatum* in the control group and the cell-free media treatments of *C. marina* var. *marina* and *G. impudicum* was higher compared to that of individual cells (Table 4). The highest growth rate of 2.61 ± 0.59 div day^−1^ was registered in the control treatment, which represents a significant difference *(p =* 5^−6^) relative to isolated chains exposed to 25 and 50 mL of cell-free media of *C. marina* var. *marina*. The generation time was significantly reduced in the control group (0.12 ± 0.02 day^−1^) relative to cells exposed to 50 mL of cell-free media of *G. impudicum* (0.11 ± 0.04 day^−1^). The generation time of isolated chains exposed to 25 and 50 mL of cell-free media of *C. marina* var. *marina* (0.22 ± 0.11 and 0.21 ± 0.13 day^−1^, respectively) was significantly higher than that in the control group; cells exposed to 25 mL of the cell-free filtrate of *G. impudicum* had a higher generation time of 0.33 ± 0.15 (*p* = 0.001) (Table 4).

## 3. Discussion

In this study, the allelopathic effect of cell-free media of *C. marina* var. *marina* and *G. impudicum* caused decreased viability, morphological changes, and an increase in the concentration of paralytic toxins in *G. catenatum*. A decrease or inhibition in the growth of the target species when exposed to allelopathic conditions has been suggested for several species under laboratory conditions [6,8,43,44]. The phytoplanktonic species *C. marina* var. *marina*, *G. impudicum*, and *M. polykrikoides* can coexist with *G. catenatum* [25,47,48,49,50,51] and cause an allelopathic effect under laboratory conditions. The allelopathic effect of cell-free media is lower in comparison with the observed effects when the species are cultivated together. Fernández-Herrera et al. [19] and Band-Schmidt et al. [17] reported growth inhibition, cell damage that promotes lysis, oxidative stress, and programmed cell death in *G. catenatum*, although they did not evaluate the response in the content of toxins, nor the viability of cells that survived the allelopathic interaction. Furthermore, the allelopathic effect differed depending on the species and the volume added to the culture of the target species [17,19]. The allelopathic effect on *G. catenatum* observed in this study does not correspond to a species-specific interaction, as it occurs with other co-occurring species because they have the same mechanism of action. Species of the *Alexandrium* genus (*A. tamarense* (Lebour) Balech, *A. ostenfeldii* (Paulsen) Balech & Tangen, *A. lusitanicum* Balech, *A. minutum* Halim, *A. catenella* (Whedon & Kofoid) Balech, and *A. taylori* (Balech) were tested on autotrophic phytoplankton species (*Rhodomonas salina*, *Dunaliella salina* (Dunal) Teodoresco, and *Thalassiosira weissflogii* (Grunow) G.A.Fryxell & Hasle), as well as on heterotrophic species (*Oxyrrhis marina* Dujardin, *Amphidinium 10rissum* Lohmann, and *Rimostrombidium caudatum* (Kahl) Agatha & Riedel-Lorjé), showing that lytic activity depends on the donor/target relationship. *Alexandrium minutum* has an allelopathic effect on *O. marina,* and *A. catenella* on *D. salina;* species-specific allelopathic interactions resulted in nine or more combinations between the tested species [45]. *Karenia brevis* (C.C.Davis) Gert Hansen & Moestrup produces multiple compounds that moderately affect the diatom *Asterionellopsis glacialis* (Castracane) [52]. However, when *Thalassiosira pseudonana* Hasle & Heimdal is exposed to *K. brevis*, the allelopathic effect promotes membrane damage, osmoregulation disturbance, and oxidative stress, contrary to the response of *A. glacialis*, which, due to the usual coexistence with *K. brevis*, has developed a partial tolerance to *K. brevis* allelochemicals [9,53]. Using transcriptomics, it has been demonstrated that the diatom *S. costatum* caused allelopathy on *K. mikimotoi*, affecting the integrity and function of the cell membrane by damaging the cell membrane structure, causing cell necrosis that led to cell death. Several metabolic processes related to ribosome and RNA transport, glycolysis/gluconeogenesis, photosynthesis, cell membrane maintenance, and osmoregulation were also affected [9].

The profile of PST detected in *G. catenatum* exposed to the allelopathic effect is similar to those found in other strains isolated from the Gulf of California [54,55]; however, the toxin concentration per cells increased. In the control treatment, the toxin concentration was 370.98 pg STXeq cell^−1^, which is more than three times higher than the highest value (101 pg STXeq cell^−1^) previously reported for *G. catenatum* strains in this region [55]. Changes in toxicity have been associated with nutrients ratios, salinity, strain, and growth stage, among other factors [56,57,58,59,60]. The allelopathic effect from the cell-free media treatments promoted an increase of as much as 836 ± 96 pg STXeq cell^−1^ in the concentration of PST. In dinoflagellate species of the genus *Alexandrium*, paralytic toxins do not have allelochemical properties [45,61,62]. The presence of predators via released molecules can induce increased toxin production in *Alexandrium* species. Selander et al. [63] showed that the copepod grazer *Acartia tonsa* Dana induces a 3.5-fold increase in PST production in *Alexandrium minutum*. The same authors [63] isolated and identified PST-inducer lipids, named copepodamides, and showed that they act in pico and nanomolar concentrations, increasing production of PST by as much as 20-fold. Another study exposing leaves of the macrophyte *Cymodocea nodosa* (Ucria) Asch to the dinoflagellate *Alexandrium pacificum* R.W.Litaker showed an increase in the production of PST toxins [64]; in agreement with results of this study, they also reported a decrease in the growth rate and morphological changes. The toxin profile detected in *A. pacificum* was Neo-STX, GTX1/3/4, and the majority of analogs were C1/2 saxitoxin analogs; 1–2 pg cell^−1^ of C1/2 analogs were detected in the control treatment, increasing to 5–6 pg cell^−1^ when *A. pacificum* was exposed to 1.5 g of leaves of *C. nodosa* [64]. Likewise, this study suggests that the allelopathic effects caused by *C. marina* var. *marina* and *G. impudicum* promote an increase in the PST concentration of *G. catenatum*. It is still necessary to identify the allelopathic metabolites present in the cell filtrates of *C. marina* var. *marina* and *G. impudicum* to demonstrate whether the responses observed in *G. catenatum* corresponds to a synchronized strategy to resist the action of allelochemicals or to a generalized adaptation to a specific metabolite. Knowing the modes of action in response to allelopathy contributes to our understanding of how species coevolved to withstand chemical pressure and interact with other phytoplankton residents during the formation, composition, and dominance of algal blooms.

The cell-free media of *C. marina* var. *marina, M. polykrikoides* and *G. impudicum* caused similar changes in *G. catenatum*, such as a decrease in growth and morphological changes [17,19]. Cell lysis caused by filtrates is a consequence of a continuous disarrangement of the cell membrane during the interaction with cell filtrates, as the first structure with which the allelochemicals have contact is the cell membrane [65,66]. Damage in the cell membrane can lead to osmotic changes, which can disrupt the life cycle of alveolates [67,68]. The internal and external changes found in *G. catenatum*, such as the loss of flagella and mobility, vacuolation, condensation of the cytoplasm with inclusion bodies, and cell elongations, are compatible with the dynamic movements of the amphiesmal arrangement, although there have been no reports of the influence of allelopathy on the amphiesmal layer. Kalinina et al. [69] and Matantseva [70] suggest that these pellicle layers are modified in response to chemical signaling of external stressors and are important in the process of cyst formation. Pozdnyakov et al. [71] studied the transcriptome of dinoflagellates and found the presence of 31 families of ion channels related to membrane potential, the calcium signaling system, translation of extracellular chemical signals, mechanical signaling, photoreception, ion transport, bioluminescence, and nutrient transport. Although the allelochemical effects of *C. marina* var. *marina* and *G. impudicum* produce on *G. catenatum* have not been characterized due to their affinity for some of the channels present in dinoflagellate membranes, it is possible that free fatty acids and reactive oxygen species are included among the synthesized allelochemicals [72]. Fatty acids, such as oleic acid (OA), linoleic acid (LA), alpha-linolenic acid (LNA), palmitic acid (PA), stearic acid (SA), eicosapentaenoic acid (EPA), docosahexaenoic acid (DHA), lauric acid (LRA), and myristic acid (MA), promote membrane disruption and cell lysis in algal and cyanobacterial species [73]. Hexadecanoic acid; 9Z, 12Z, and 15Z octadecatrienoic acids; and the 9E octadecenoic acid extracted from the macroalga *Ulva linza* have an allelopathic effect on the microalga *Platymonas helgolandica (=Tetraselmis helgolandica* (Kylin) Butcher) and the dinoflagellate *Prorocentrum minimum* (Pavillard) J.Schiller [74]. The early signs of cell membrane damage caused by cell filtrates from *C. marina* var. *marina* and *G. impudicum* in *G. catenatum* can result in osmotic degradation of the protoplast and cell organelles, which lose their integrity and function of the ion channels, triggering electrical potential differences between the inside and outside of cells.

Band-Schmidt et al. [75] reported the average cell size of seven strains isolated from Bahía Concepción, Gulf of California. Live single cells had a width ranging from 25.04 to 54.73 μm and a length ranging from 37.83 to 69.99 μm. Live four-cell chains were slightly smaller, measuring in the range of 30.18 to 44.5 μm wide and 32.75 to 57.38 μm long. These sizes are within the average measurements of the strain GCBAPAZ-10 isolated from Bahía de La Paz used in the present study, with a width ranging from 34 to 54 μm, a length ranging from 34 to 67 μm, and a volume of 19,550 μm^3^. These average values were obtained from single cells and chain-forming cells. Larger *G. catenatum* cells were found when exposed to cell filtrates of *C. marina* and *G. impudicum*, with an average width of 28–66 μm, a length of 30–82 μm, and a cell volume of 27,765 μm^3^. These cells are also larger (width, 22–33 μm; length, 30–46 μm; cell volume, 18,750 μm^3^) than those reported from other geographical regions [76,77,78,79]; however, these smaller sizes could be due to the effect of the measurements of living and fixed cells (Table S3). These results indicate that the allelopathic effect promotes increased size in *G. catenatum*.

Shapes and sizes similar to those we found due to the allelopathic effect have only been reported when the process of sexual reproduction occurs. Blackburn et al. [77] and Figueroa et al. [79,80] reported that the size range of a planozygote is between 31 and 59 μm wide and 53 to 84 μm long; such cells are also characterized by two flagella, products of the fusion of gametes. In the allelopathic treatments in the present study, no biflagellate *G. catenatum* cells were observed. Bravo and Figueroa [81] described that within the evolutionary mechanism of dinoflagellates, one of the strategies employed in their life cycle is the formation of temporary and resistance cysts. Additionally, factors such as habitat, temperature, light, availability of nutrients, predation, and parasitism influence their formation [77,79,80,81]. Results from this study suggest that cell-free media of *C. marina* var. *marina* and *G. impudicum* promote the formation of temporary cysts in *G. catenatum* as a survival strategy against allelopathy; this has also been observed in the dinoflagellates *Scrippsiella trochoidea* [10,45] and *Kryptoperidinium foliaceum* (F.Stein) Lindemann, (Hakanen et al. [82]), except that in the experiments in the present study, none of the reisolated temporal cysts were viable.

Hakanen et al. [82] suggested that in nature, the plankton community may be able to better resist the allelopathic effects of other species, a situation that does not occur when allelopathy is demonstrated under laboratory conditions [41,53]. It is likely that in the natural environment, cells and cysts of *G. catenatum* exposed to the allelopathic effect of donor species may exhibit increased viability when migrating through the water column and moving away from the influence of allelochemicals, which does not occur when confined to a reduced space under experimental conditions. Shang et al. [28] reported that *Alexandrium leei* promotes the separation of four-cell chains of *M. polykrikoides* into individual cells as a consequence of the allelopathic effect; the authors suggested that chain forming or separation into single cells in species that employ this strategy confers an advantage with respect to survival of allelopathic agents. Results from this study suggest that *G. catenatum* could form chains to survive the allelopathic effect, as chain-forming cells are more likely to have biologically viable cells and owing to their ability to perform vertical migrations [83] through the water column, which would facilitate their movement away from the influence of allelochemicals.

## 4. Conclusions

The allelopathic effect caused changes in the concentration of paralytic toxins, and the toxin profile exhibited an increased concentration of less potent saxitoxin analogs. *Gymnodinium catenatum* formed non-viable temporary cysts after being isolated from the cell-free media of *C. marina* and *G. impudicum*, and single cells were more sensitive to the allelopathic effect in comparison to chain-forming cells. Individual and chain-forming cells exposed to the cell-free filtrates of *C. marina* and *G. impudicum* exhibited reduced cell viability, growth rate, and number of generations per day. Furthermore, the allelopathic effect promoted by the cell-free media of *Chattonella marina* var. *marina* and *Gymnodinium impudicum* in *Gymnodinium catenatum* caused morphological changes and an increase in volume, leading to cell lysis. Our results suggest that *G. catenatum* uses mobility through chain-forming cells and cyst formation as strategies to survive allelopathy, in addition to increasing the toxin content.

## 5. Materials and Methods

### 5.1. Allelopathy Experiment: Changes in Cell Shape and Volume

Isolated strains from the Gulf of California, *Chattonella marina* var. *marina* (CMCV-2), *Gymnodinium catenatum* (GCBAPAZ-10), and *Gymnodinium impudicum* (GIBACO-13), were used in the present study [17,84]. Batch cultures of each strain were cultured in 1 L flasks with 500 mL of modified GSe medium [85] at an initial cell density of 1000 cells mL^−1^ in culture conditions of 1:12 h light:dark cycle, ~150 µmol photons m^−1^ s^−1^ of irradiance at 24 ± 1 °C, and salinity of 34‰. All experiments were carried out under the same conditions. During the early exponential phase, cells from *C. marina* var. *marina* and *G. impudicum* were removed by gentle filtration through fiberglass GF/F filters (Whatman^®^, Canterbury, UK). Cell-free filtrates (25 and 50 mL) of *C. marina* var. *marina* and *G. impudicum* were added to *G. catenatum* cultures in 300 mL flasks with a volume of 150 mL; volume proportions are shown in Table S4. As controls, two treatments of *G. catenatum* were inoculated with 25 and 50 mL of their own cell-free media. After 48 h, 2 mL samples were fixed with Lugol to determine cell abundance on a 1.0 mL Sedgwick-Rafter counting chamber under an inverted microscope (Carl Zeiss, Oberkochen, Germany, Axio Vert. A1). All treatments were performed in triplicate.

Morphological changes were evaluated in live cells of *G. catenatum.* The volume (V) was measured in *G. catenatum* cells (*n* = 30) of each treatment, assuming the shape of an ellipsoidal sphere, based on measurements of the (b) length and (c) width in the cross section, as well as the (a) length of the transapical section using the formula suggested in [86] (Figure S2).

(1) $$V=\;\frac{\pi }{6}\;\cdot \;a\;\cdot \;b\;\cdot \;c$$

### 5.2. Paralytic Toxin Profile

After 48 h of exposure to cell-free media, for PST determination, an aliquot of 50 mL of *G. catenatum* was concentrated by filtration using glass GF/F filters (Whatman^®^, Canterbury, UK) and preserved at −20 °C until further analysis. The filters were deposited in a microcentrifuge tube, and extraction was carried out by maceration with the addition of 2 mL of acetic acid (0.03 N). The supernatant was transferred to a 25 mL tube and sonicated at (35 kHz) for 5 min. Tubes were centrifuged at 5700× *g* for 15 min at 15 °C. Subsequently, the supernatant was filtered through acrodisks of 13 mm and 0.45 μm, a 150 μL aliquot of the extract was hydrolyzed with HCl (1M), and N-sulfocarbamated toxins (GTX5/B1 and GTX6/B2) were quantified in relation to peaks of carbamated toxins formed during HCl treatment (B1 to STX, B2 to neoSTX, C1 to GTX2, C2 to GTX3, C3 to GTX1, and C4 to GTX4). A 20 μL sample was injected into the HPLC system (HP 1360 Infinity II) with a fluorescence detector (HP 1116) wavelengths (ʎ) of 330 nm (excitation) and 395 nm (emission) following the methods described in [87] and [88], respectively. Toxins were identified and quantified using reference standards from the National Research Council, Canada. Limits of detection and limits of quantification are listed in Table S5. The structures of the saxitoxin analogs were visualized using ACD/ChemSketch 12.01 software (Advance Chemistry Development, Inc., Toronto, ON, Canada).

### 5.3. Viability, Growth Rate, and Generation Time of Single and Four-Cell Chains of G. catenatum exposed to Allelochemicals

After 48 h of exposure to medium containing allelochemicals, single and four-cell chains of *G. catenatum* were reisolated with capillary micropipettes into 96-well plates containing 135 µL of GSe medium in each well to determine their cell viability. Single cells and four cell-chains (*n* = 50 of each) of *C. marina* var. *marina* and *G. impudicum* from the control and treatment groups exposed to cell-free media (25 and 50 mL) were reisolated. The percentage of viability (*Pv*) was calculated following the formula proposed in [89] and used in [90] to determine reisolated post-shorting viability, adapted to micropipette single-cells.

(2) Pv=(Vw) (100%)/Vw cont

where the number of wells with growth viability (*Vw*) with cells of *G. catenatum* exposed to cell-free media is related to the number of wells with growth viability cells in the control (*Vw cont*) 192 h after reisolation.

Cell density in each well was used to calculate growth rates (µ) [91] and to determine the number of generations per day (tg) [92] at 96 and 192 h.

### 5.4. Statistical Analysis

Data are presented as mean ± SD (SD), and Kolmogorov–Smirnov and Shapiro–Wilk normality tests and the Levene test for homoscedasticity were run. For normal data, one-way analysis of variance (ANOVA) with Tukey’s *post hoc* comparison tests were applied to determine differences between treatments (*p* ≤ 0.05). For data that did not follow a normal distribution, Kruskal–Wallis tests for comparison of multiple independent samples (*p* ≤ 0.05) were applied. All statistical analyses were run using Statistica StatSoft^®^ software (Tulsa, OK, USA).

## Figures and Tables

**Table 1:** Volume of *Gymnodinium catenatum* after 48 h of exposure to cell filtrates of *Chattonella marina* var. *marina* and *Gymnodinium impudicum*.

Treatment	(*n*)	Abundance (Cells mL^−1^)	Width (µm)	Length (µm)	Biovolumen (µm^3^)
Control	30	1966 ± 95	43.95 ± 5.55	49.08 ± 8.37	19,550 ± 8316 ^a^
Cell-free media of *Chattonella marina* var. *marina*					
25 mL	30	902 ± 363	46.58 ± 10.91	52.04 ± 15.38	27,630 ± 19,520 ^b^
50 mL	30	860 ± 79	45.34 ± 0.99	51.91 ± 15.78	27,502 ± 15,636 ^b^
Cell-free media of *Gymnodinium impudicum*					
25 mL	30	1268 ± 316	46.43 ± 11.01	53.30 ± 17.39	24,425 ± 7628 ^a^
50 mL	30	1229 ± 370	46.37 ± 10.78	52.17 ± 21.54	27,505 ± 2231 ^a^

Data are show as mean ± SD. Different letters indicate significant differences between the treatments, and the same letters indicate no statistical differences (one-way ANOVA, *p* < 0.05).

**Table 2:** Average toxin profile (% mol) of *Gymnodinium catenatum* exposed to cell-free media of *C. marina* and *G. impudicum*.

Treatment	GTX2/3	dcSTX	dcGTX2/3	B 1	C 1/2
Control	1.43 ± 0.26 ^a^	0.67 ± 0.03 ^a^	3.33 ± 0.21 ^a^	0.09 ± 0.01 ^a^	94.44 ± 0.48 ^a^
Cell-free media of *Chattonella marina* var. *marina* 25 mL	5.6 ± 0.45 ^a^	1.58 ± 0.01 ^b^	7.0 ± 0.43 ^b,^*	0.53 ±0.09 ^b^	85.84 ± 3.46 ^b^
50 mL	1.48 ± 0.06 ^a^	0.61 ± 0.08 ^a^	2.91 ± 0.16 ^c^	0.04 ±0.02 ^a^	94.96 ± 0.11 ^a^
Cell-free media of *Gymnodinium impudicum* 25 mL	1.40 ± 0.02 ^a^	0.54 ± 0.11 ^a^	2.55 ± 0.28 ^a^	0.08 ±0.06 ^a^	95.42 ± 0.37 ^a^
50 mL	1.62 ± 0.03 ^a^	0.48 ± 0.04 ^a^	2.25 ± 0.07 ^a^	0.12 ±0.02 ^a^	95.52 ± 0.06 ^a^

(* dcGTX3 relative to detection limit). Different letters indicate significant differences between the treatments; the same letters indicate no statistical differences (Kruskal–Wallis test, *p* < 0.05). STX, Neo, GTX1, GTX4, dcNeo, B2, C3, and C4 were not detected.

**Table 3:** Cell abundance, growth rate, generation time, and viability of single *Gymnodinium catenatum* cells reisolated after exposure to cell-free filtrates of *Chattonella marina* var. *marina* and *Gymnodinium impudicum*.

Treatment	Abundance after 96 h (Cells)	Abundance after 192 h (Cells)	Growth Rate (Div Day^−1^)	Generations Day^−1^	*Pv* Viability (%)	*Pv* Relative to Control (%)
Control	3 ± 1	5 ± 2	1.57 ± 0.38 ^a^	0.14 ± 0.03 ^a^	46 ± 3 ^a^	-
Cell-free media of *Chattonella marina* var. *marina*						
25 mL	2 ± 2	3 ± 1	0.55 ± 0.33 ^b^	0.16 ± 0.02 ^a^	14 ± 4 ^b^	−69.6 ± 2
50 mL	1 ± 1	4 ± 3	1.03 ± 0.49 ^a^	0.32 ± 0.15 ^b^	8 ± 3 ^b^	−82.6 ± 2
Cell-free media of *Gymnodinium impudicum*						
25 mL	2 ± 1	3 ± 2	0.45 ± 0.33 ^b^	0.33 ± 0.15 ^b^	24 ± 6 ^c^	−47.9 ± 6
50 mL	2 ± 1	3 ± 1	0.90 ± 0.17 ^a^	0.31 ± 0.02 ^b^	20 ± 2 ^c^	−56.6 ± 3

Percentage viability (***Pv***). Data are shown as mean ± SD. Different letters indicate significant differences between the treatments, and the same letters indicate no statistical differences (Kruskal–Wallis test, *p* < 0.05).

**Table 4:** Cell abundance, growth rate, generation time, and viability of four-cell chains of *Gymnodinium catenatum* reisolated after exposure to cell-free media of *Chattonella marina* var. *marina* and *Gymnodinium impudicum*.

Treatment	Abundance after 96 h (Cells)	Abundance after 192 h (Cells)	Growth Rate (Div Day^−1^)	Generations Day^−1^	*Pv* Viability (%)	*Pv* Relative to Control (%)
Control	6 ± 2	35 ± 10	2.61 ± 0.59 ^a^	0.12 ± 0.02 ^a^	62 ± 3 ^a^	-
Cell-free media of *Chattonella marina* var. *marina*						
25 mL	2 ± 1	3 ± 1	1.53 ± 0.53 ^b^	0.22 ± 0.11 ^b^	54 ± 12 ^a^	−13 ± 1
50 mL	2 ± 1	8 ± 2	1.23 ± 0.21 ^b^	0.21 ± 0.13 ^b^	38 ± 2 ^b^	−54.9 ± 4
Cell-free media of *Gymnodinium impudicum*						
25 mL	6 ± 1	15 ± 4	1.97 ± 0.43 ^a^	0.33 ± 0.15 ^c^	50 ± 7 ^a^	−50.1 ± 2
50 mL	5 ± 2	4 ± 1	1.75 ± 0.48 ^a^	0.11 ± 0.04 ^a^	41 ± 9 ^c^	−33.9 ± 3

Percentage viability (***Pv***). Data are show as mean ± SD. Different letters indicate significant differences between the treatments, and the same letters indicate no statistical differences (Kruskal–Wallis test, *p* < 0.05).

## Data Availability

Not applicable.

## Supplementary Materials

The following supporting information can be downloaded at: https://www.mdpi.com/article/10.3390/toxins14090616/s1, Figure S1: Percentage of *Gymnodinium catenatum* single cells and 2, 4 and 8 cell chains after exposure to free-culture media from *Chattonella marina* var. *marina* and *Gymnodinium impudicum*; Figure S2: Measurements in *Gymnodinium catenatum* cells assuming the shape of an ellipsoid; Video S1. Changes in the morphology of *G. catenatum*, such as rounded cells with multiple vacuolation, condensation of the cytoplasm with accumulation body; Table S1. Toxic content per type of saxitoxin analogs in *Gymnodinium catenatum* after exposure to cell-free media; Table S2. Average (% mol) by saxitoxin analogs in *Gymnodinium catenatum* after exposure to cell-free media; Table S3: Cell size of vegetative cells of *Gymnodinium catenatum* from different geographical regions; Table S4: Proportion of cell-free media aggregated to the treatments in allelopathic experiments of *Gymnodinium catenatum*; Table S5. STX, saxitoxin; dcSTX, decarbamoyl STX, NeoSTX, neosaxitoxin; GTX, gonyautoxin; dcGTX, decarbamoyl gonyautoxin; dcNeo, decarbamoyl neosaxitoxin. LOD, Limit of detection; LOQ, limit of quantification.
