# Supplementary material for: Changes in Toxin Production, Morphology and Viability of Gymnodinium catenatum Associated with Allelopathy of Chattonella marina var. marina and Gymnodinium impudicum
Source: Toxins (Basel). 2022 Sep 3;14(9):616. doi: 10.3390/toxins14090616 (PMC9505736; doi:10.3390/toxins14090616)
Supplement: Supplementary file 1 [file toxins-14-00616-s001.zip › toxins-1874016-supplementary/Suppementary material 1874016/Fernandez-Herrera et al 2022_Supplementary_Material_Toxins.pdf]

toxins

Supplementary Material

Changes in Toxin Production, Morphology and Viability of *Gymnodinium catenatum* Associated with Allelopathy of *Chattonella marina* var. *marina* and *Gymnodinium impudicum*

Leyberth José Fernández-Herrera, Christine Johanna Band-Schmidt, Tania Zenteno-Savín, Ignacio Leyva-Valencia, Claudia Judith Hernández-Guerrero, Francisco Eduardo Hernández-Sandoval and José Jesús Bustillos-Guzmán

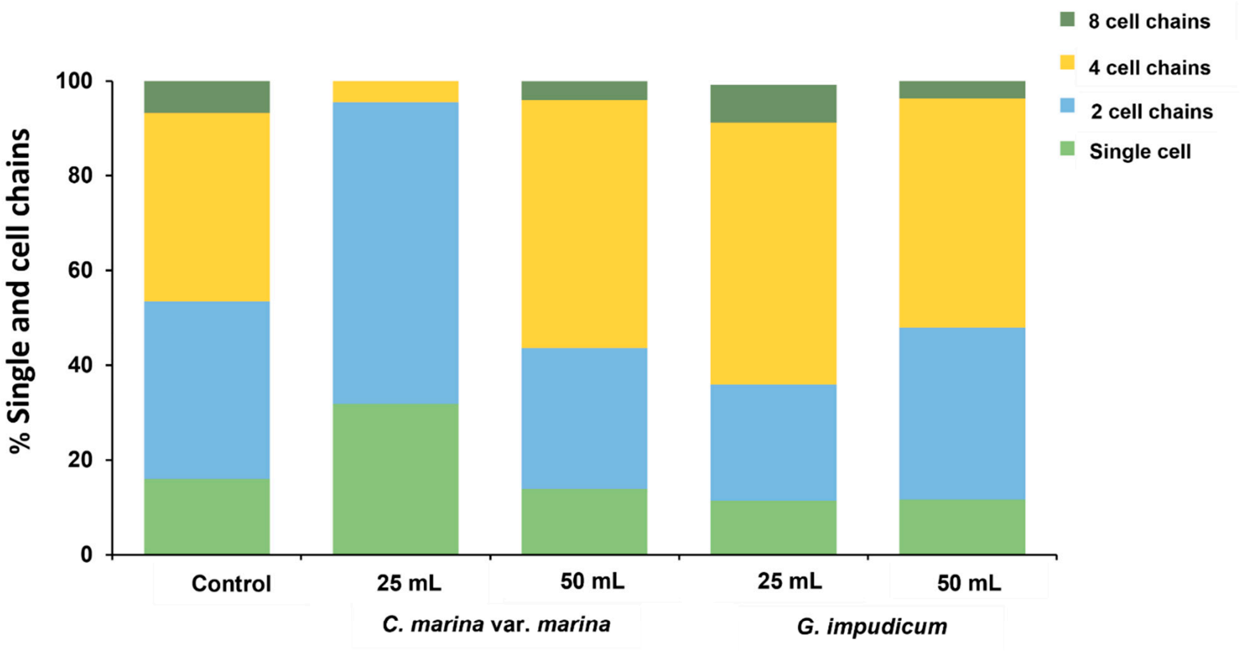

Treatments	Single cell (%)	2 cell chains (%)	4 cell chains (%)	8 cell chains (%)
Control	15.98 ± 2.65 ^{a}	37.46 ± 2.00 ^{a}	39.79 ± 4.47 ^{a}	6.77 ± 0.16 ^{a}
C. marina var. *marina* 25 mL	31.82 ± 10.84 ^{b}	63.64 ± 17.65 ^{b}	4.55 ± 0.60 ^{b}	-
C. marina var. *marina* 50 mL	13.85 ± 2.50 ^{a}	29.72 ± 9.54 ^{a}	52.32 ± 10.98 ^{c}	4.03 ± 1.64 ^{b}
G. Impudicum 25 mL	11.32 ± 1.34 ^{a}	24.56 ± 10.66 ^{a}	55.31 ± 7.96 ^{c}	8.02 ± 4.77 ^{a}
G. Impudicum 50 mL	11.63 ± 1.24 ^{a}	36.28 ± 4.66 ^{a}	48.37 ± 4.33 ^{c}	3.72 ± 1.73 ^{b}

Figure S1: Percentage of *Gymnodinium catenatum* single cells and 2, 4 and 8 cell chains after exposure to free-culture media from *Chattonella marina* var. *marina* and *Gymnodinium impudicum*. Data are shown as mean ± standard deviation. Different letters indicate significant differences between treatments, the same letters indicate no statistical differences (One-way ANOVA, $p < 0.05$).

The percentage value of the proportion added was adjusted to a final volume of 150 mL and 1,000 cells mL^{-1} of *G. catenatum*.

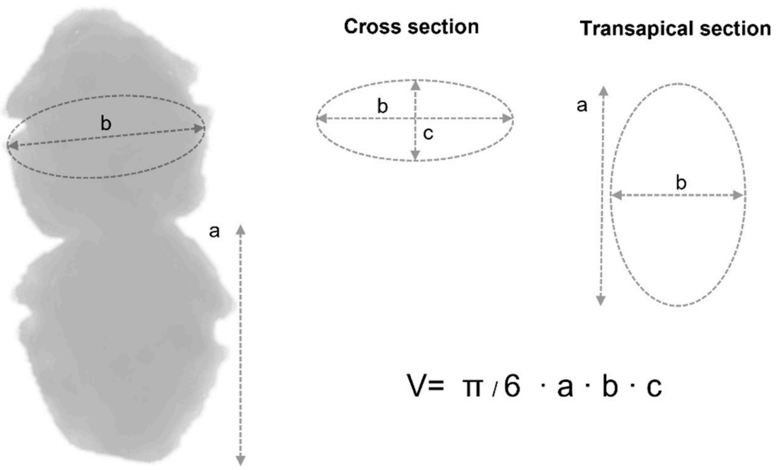

Figure S2: Measurements in *Gymnodinium catenatum* cells assuming the shape of an ellipsoid, based on length and width measurements of the (b) length and (c) width in the cross section, and (a) length and width (b) in the transapical section (Sun and Lu, 2013).Measurement pattern in *Gymnodinium catenatum* cells assuming the shape of an ellipsoidal, based on length and width measurements of the (b) length and (c) width in the cross section, and (a) length and width (b) in the transapical section (Sun and Lu, 2013).

Table S1. Toxic content per type of saxitoxin analogs in *Gymnodinium catenatum* after exposure to cell-free media from *Chattonella marina* var. *marina* and *Gymnodinium impudicum* in allelopathy experiments.

**Treatments**	**Sulfocarbamoyl B1, C1/2 (pg cell^{-1})**	**Decarbamoyl dcSXT, dcGTX2/3 (pg cell^{-1})**	**Carbamoyl GTX2/3 (pg cell^{-1})**
Control	356.08 ± 39.58 [a]	10.63 ± 0.79 [a]	4.27 ± 0.21 [a]
Chattonella marina var. *marina*			
25 mL	24.08 ± 4.11 [b]	1.55 ± 0.24 [b]	0.51 ± 0.07 [b]
50 mL	802.12 ± 10.30 [c]	22.56 ± 1.78 [c]	12.03 ± 0.02 [c]
Gymnodinium impudicum			
25 mL	670.89 ± 8.76 [c]	15.31 ± 0.02 [a]	8.16 ± 0.30 [c]
50 mL	702.30 ± 25.15 [c]	14.20 ± 0.32 [a]	9.90 ± 0.67 [c]

Data are show as mean ± standard deviation. Different letters indicate significant differences between treatments, the same letters indicate no statistical differences (Kruskal–Wallis test, $p < 0.05$).

Table S2. Average (% mol) by saxitoxin analogs in *Gymnodinium catenatum* after exposure to cell-free media from *Chattonella marina* var. *marina* and *Gymnodinium impudicum* in allelopathy experiments.

**Treatments**	**Sulfocarbamoyl B1, C1/2 (%)**	**Decarbamoyl dcSXT, dcGTX2/3 (%)**	**Carbamoyl GTX2/3 (%)**
Control	94.44 ± 0.48	3.38 ± 0.21	1.43 ± 0.26
Chattonella marina var. *marina*			
25 mL	85.84 ± 3.86	7 ± 0.43	5.06 ± 3.41
50 mL	94.96 ± 0.11	2.91 ± 0.16	1.48 ± 0.06
Gymnodinium impudicum			
25 mL	95.43 ± 0.37	2.55 ± 0.28	1.40 ± 0.02
50 mL	95.52 ± 0.06	2.26 ± 0.07	1.62 ± 0.03

Data are show as mean ± standard deviation. No statistically significant differences were found between treatments (Kruskal–Wallis test, $p < 0.05$).

Table S3: Cell size of vegetative cells of *Gymnodinium catenatum* from different geographical regions.

Live / fixed cells	Width (μm)	Length (μm)	Volume (μm^3)	Origen	Reference
Fixed	22 - 33	30 - 46	-	Gulf of California	Graham, 1943
Live-fixed	30 – 43	48 – 65	-	Japan	Yuki and Yushimatsu, 1987
Live	27 – 36	23 – 41	-	Tasmania	Blackburn et al., 1989
Live-fixed	25.04 – 54.73	37.83 – 69.99	-	Pacific coast and Gulf of California, Mexico	Band-Schmidt et al., 2008
Fixed	38 ± 7	38 ± 8	-	Atlantic coast, Spain	Figueroa et al., 2008
Live	35.6 – 52. 1	48.2 – 66.5	-	China	Gu et al., 2013
Fixed	20	45	18,750	Pakistan	Munir et al., 2015
Live	24 – 54	34 – 67	19,550	Gulf of California, Mexico	This study

(-) no measurement.

Table S4: Proportion of cell-free media aggregated to the treatments in allelopathic experiments of *Gymnodinium catenatum*.

Treatment	Cell-free media (%)	GSe media (%)	*Gymnodinium catenatum* culture (%)
Gymnodinium catenatum			
Control 25 mL	15	70	15
Control 50 mL	30	50	20
Chattonella marina var. *marina*			
25 mL	15	70	15
50 mL	30	50	20
Gymnodinium impudicum			
25 mL	15	70	15
50 mL	30	50	20

Table S5. STX, saxitoxin; dcSTX, decarbamoyl STX, NeoSTX, neosaxitoxin; GTX, gonyautoxin; dcGTX, decarbamoyl gonyautoxin; dcNeo, decarbamoyl neosaxitoxin. LOD, Limit of detection; LOQ, limit of quantification

**Toxin**	**LOD (ng/mL)**	**LOQ (ng/mL)**
STX	6.69	22.31
dcSTX	14.10	47.02
NeoSTX	1119.15	3730.50
GTX 2	37.16	123.88
GTX 3	42.03	140.11
dcGTX 2	48.28	160.95
dcGTX 3	45.89	152.97
dcNeo	37.16	123.88
